# Contribution of health history and neuropathologic changes to the likelihood of dementia in those with intermediate/high Alzheimer’s pathology: findings from* The 90 + Study*

**DOI:** 10.1007/s00401-026-03010-9

**Published:** 2026-04-06

**Authors:** Zarui A. Melikyan, Zeinah Al-darsani, Luohua Jiang, Katherine A. Colcord, Annlia Paganini-Hill, Syed A. Bukhari, Thomas J. Montine, Claudia H. Kawas, María M. Corrada

**Affiliations:** 1 Department of Neurobiology and Behavior, University of California, 1400 Bio Sci III, Irvine, CA 92697 USA; 2 Department of Epidemiology and Biostatistics, Temple University College of Public Health, 1210 W. Berks Street, 3Rd Floor Paley Hall, Philadelphia, PA 19122 USA; 3 Department of Epidemiology and Biostatistics, University of California, 856 Health Sciences Road, Suite 3400, Irvine, CA 92617 USA; 4 Institute for Memory Impairments and Neurological Disorders, University of California, 2642 Biological Sciences III, Irvine, CA 92697 USA; 5 Beckman Laser Institute & Medical Clinic, University of California, 1002 Health Sciences Rd, Irvine, CA 92612 USA; 6 Department of Pathology, Stanford University School of Medicine, Lane Building300 Pasteur Drive, Stanford, CA L23594305 USA; 7 Department of Neurology, University of California, 101 The City Drive, IrvineOrange, CA 92868 USA

**Keywords:** Oldest old, Neuropathologic change, Non-ADNC, Dementia vs. normal cognition in the presence of intermediate/high ADNC

## Abstract

Although intermediate/high Alzheimer’s Disease Neuropathologic Change (ADNC) is associated with dementia in many older adults, some remain cognitively normal and are often referred to as resilient to ADNC. We aim to examine health, lifestyle, and neuropathologic factors that distinguish older adults with dementia vs. normal cognition in the presence of intermediate/high ADNC. Participants were from *The 90* + *Study*, a longitudinal study of aging in southern California. This cross-sectional analysis included participants with an intermediate/high ADNC on neuropathologic exam and normal cognition or dementia diagnosis on case conference. We analyzed 11 neuropathologic changes, both vascular and neurodegenerative, dichotomized as present/absent, and the total number of neuropathologies. To examine the association of health and lifestyle factors and neuropathologic changes (predictors) with cognitive diagnosis at consensus case conference, dementia vs. normal cognition, (outcome), we used logistic regression adjusted for demographics. Among 235 participants (mean age at death = 98 years, 70% women), 33% maintained normal cognition. Participants with heart disease (OR = 0.45; 95% CI = 0.25, 0.81) and hypertension (OR = 0.53; 95% CI = 0.29, 0.95) had lower likelihood of dementia. In contrast, participants with a history of transient ischemic attacks (OR = 3.00; 95% CI = 1.46, 6.18), Lewy Body Disease (OR = 2.73; 95% CI = 1.14, 6.58), hippocampal sclerosis (OR = 2.70; 95% CI = 1.06, 6.86), Limbic-predominant Age-related TDP-43 Encephalopathy neuropathologic change (OR = 2.80; 95% CI = 1.53, 5.12), and a high number of non-ADNCs (OR = 4.46; 95% CI = 2.01, 9.92) had higher likelihood of dementia. Arteriolosclerosis, atherosclerosis, cerebral amyloid angiopathy, and microvascular lesions were not associated with dementia. In this study, the presence of neurodegenerative neuropathologic changes other than ADNC and the absence of hypertension distinguish oldest old individuals with dementia from those with normal cognition. Understanding mechanisms underlying normal cognition in those with ADNC may provide important clues to prevention and resilience to the effects of AD neuropathology.

**Supplementary Information:**

The online version contains supplementary material available at 10.1007/s00401-026-03010-9.

## Introduction

Alzheimer’s Disease Neuropathologic Change (ADNC) is one of the most common age-related neuropathologic changes [30, 44], and while many older adults with high levels of ADNC develop dementia, some remain cognitively normal until death [1, 2, 7]. Yet, factors that distinguish older adults with dementia from those with normal cognition in the context of high ADNC levels, are not well understood. Specifically, only two studies have compared older adults with dementia (referred to as cognitively non-resilient to ADNC) to those with normal cognition (referred to as cognitively resilient to ADNC) in the presence of moderate/severe ADNC. One of these studies has defined resilience as normal cognition and moderate/high ADNC, and found that lower severity of Limbic-predominant age-related TDP-43 encephalopathy neuropathologic change (LATE-NC) and Lewy Body Disease (LBD), and presence of arteriosclerosis were associated with higher likelihood of resilience vs. non-resilience [12]. The other study, using a more restrictive definition of resilience—normal cognition in the presence of severe ADNC and no other primary neuropathology—has found that lower body mass index, smoking, and use of antiplatelet/anticoagulant agents, but not neuropathologic changes, were associated with higher likelihood of cognitive resilience [1]. By contrast, other studies [2, 45], including one from our group [35], have compared older adults with and without dementia in the presence of high ADNC levels, and did find the association of cognitive resilience to ADNC with lower levels of neuropathologic changes: cortical phosphorylated tau, fewer neocortical AD lesions, no or low levels of non-ADNCs: HS, LATE-NC, cortical aging-related tau astrogliopathy (ARTAG), LBD, cerebrovascular disease, and microinfarcts [35].

In this study, we provide additional evidence on the factors that distinguish individuals with dementia from those with normal cognition in the presence of intermediate/high ADNC, to clarify and reconcile the somewhat discordant reports in the literature. Specifically, the aims of this study were to identify: (1) health and lifestyle factors and (2) neuropathologic changes that distinguish older adults with dementia from those with normal cognition in the presence of intermediate/high ADNC.

## Materials and methods

### Study design

***The 90* + *Study*** is an ongoing longitudinal study of aging and cognition of persons aged 90 + years. Participants of ***The 90* + *Study*** include (1) survivors aged 90 years and older of the Leisure World Cohort Study (LWCS), a population-based study of residents of a Southern California retirement community, who answered a postal health and lifestyle questionnaire in the 1980s [34], and (2) volunteer residents of Orange County, California aged 90 + years who are able to provide informed consent to complete study procedures [28]. While LWCS participants were recruited regardless of cognitive status, volunteers had no or mild dementia at enrollment.

### Data collection

***The 90* + *Study*** participants undergo comprehensive evaluations every 6 months that include self-reported medical history and lifestyle factors, neurological examination, and neuropsychological testing [27]. To minimize missing data, we visit participants in their homes, modify evaluations to accommodate their diminished sensory capacities, and keep the evaluations relatively short [9].

*Health history and lifestyle factors* Health history, operationalized as ever or never having reported a medical condition, includes diabetes, heart disease, hypercholesterolemia, hypertension, head trauma, Parkinson’s disease, seizures, stroke, transient ischemic attack (TIA), cataracts, rheumatoid arthritis, cancer, thyroid disease, and body mass index calculated from height and weight measured at the visit (continuous). We defined heart disease as having any of the following: coronary artery disease, myocardial infarction, arrhythmia, heart valve disease, congestive heart failure, coronary bypass surgery, or pacemaker. DNA for Apolipoprotein ε (APOE) genotyping was obtained from either blood or a cheek swab. An APOE ε4 carrier was defined as having at least one ε4 allele, and an APOE ε2 carrier was defined as having at least one ε2 allele. Lifestyle factors included ever or never smoking and alcohol use.

*Cognitive diagnosis at consensus case conference* After death, a final cognitive diagnosis is assigned at a multidisciplinary consensus case conference using all available information from the longitudinal evaluations, as well as medical records and clinical brain imaging, when available. Neuropsychological test scores are compared against age-adjusted norms developed for the oldest old [27]. All cognitive diagnosis assignments are blinded to neuropathologic evaluation results. Participants are diagnosed as having dementia, Cognitive Impairment No Dementia (CIND), or normal cognition. Dementia is assigned when there is an impairment in at least two cognitive domains and an inability to perform at least one instrumental activity of daily living (IADL) [3]. CIND is assigned when there is an impairment in (1) a single domain of memory, or (2) any two cognitive domains, or (3) any single cognitive domain and difficulty performing one IADL, but the criteria for dementia are not met [14]. Normal cognition is assigned when there is no substantial impairment on any cognitive domain (i.e., test scores do not indicate an impairment on a cognitive domain) and no functional difficulties due to cognitive loss. Participants with CIND were not included in the analyses because they may represent a prodromal dementia stage.

*Neuropathologic evaluation* A subset of participants consented to brain autopsy. Brains were procured by the University of California Irvine (UCI) Alzheimer’s Disease Research Center (ADRC) Neuropathology Core, fixed in formalin, and sent to the Department of Pathology at Stanford University, where pathological evaluations were performed with current consensus criteria, blinded to participant information. We considered 11 neuropathologic changes: 7 neurodegenerative and 4 vascular. The seven neurodegenerative neuropathologic changes include: (1) ADNC (none, low, intermediate, high) based on the National Institute on Aging-Alzheimer’s Association (NIA-AA) ‘ABC’ score, which incorporates Thal phase for beta amyloid (Aβ) plaques, Braak staging for neurofibrillary tangles (NFT), and Consortium to Establish a Registry for AD (CERAD) staging for neuritic plaques [31]. (2) Thal phase (0–5) for Aβ senile plaques which are extracellular deposits of Aβ peptides [31, 41], (3) Braak stage (0–VI) which reflects intraneuronal deposits of misfolded tau protein [8, 31], (4) CERAD stage (none, sparse, moderate, frequent) which reflects neuritic plaques (a subset of senile plaques) closely associated with neuronal injury [29, 31], (5) Lewy body disease (LBD) (none, brainstem, amygdala, limbic, neocortical), accumulation of aggregated α-synuclein into Lewy bodies and Lewy neurites in neurons and neuronal processes [26, 31], (6) HS (present, absent) which is neuronal loss and gliosis primarily in the CA1 hippocampal subfield and subiculum, with relative sparing of the CA2–CA4 hippocampal subfields [17] (7) LATE-NC (none, amygdala, hippocampus, frontal cortex), a transactive response DNA binding protein of 43kDA proteinopathy commonly observed past age 80 years in limbic brain structures [32]. The four vascular neuropathologic changes include: (1) arteriolosclerosis (none, mild, moderate, severe), a pathologic thickening of arteriolar walls [38], (2) atherosclerosis (none, mild, moderate, severe), a buildup of plaque in the arteries of the brain [21], (3) cerebral amyloid angiopathy (CAA) (none, mild, moderate, severe), an accumulation of amyloid beta peptide within the small to medium-sized cerebral blood vessels [43], (4) microvascular lesions, microscopic infarcts or hemorrhages [31].

We dichotomized variables for ease of interpretation, and we did so by grouping together categories that had similar associations with dementia in this cohort, whenever possible given the distributions, consistent with our previous work [33]. Variables were dichotomized as follows: Thal phase (1–3 vs. 4–5), Braak stage (III–IV vs. V–VI), CERAD score (none, sparse, moderate vs. frequent), LBD (none, brainstem, amygdala vs. limbic, neocortical), LATE-NC (none vs. amygdala, hippocampus, frontal cortex), arteriolosclerosis (none vs. mild, moderate, severe), atherosclerosis (none, mild, moderate vs. severe), CAA (none vs. mild, moderate, severe), microvascular lesions (0–2 vs. 3 +). Due to the sparse numbers of observations in the most severe categories for individuals with normal cognition, we used another way to dichotomize arteriolosclerosis (none, mild vs. moderate, severe), atherosclerosis (none, mild vs. moderate, severe), and MVL (0–1 vs. 2 +). Additionally, we calculated the total number of non-ADNCs by summing the dichotomized HS, LBD, LATE, arteriolosclerosis, atherosclerosis, CAA, and MVL. We subsequently grouped the total number of non-ADNCs into three categories (0–1, 2, and 3–6) to achieve similar numbers of participants with and without resilience in each category.

### Participants

**Fig. 1:**
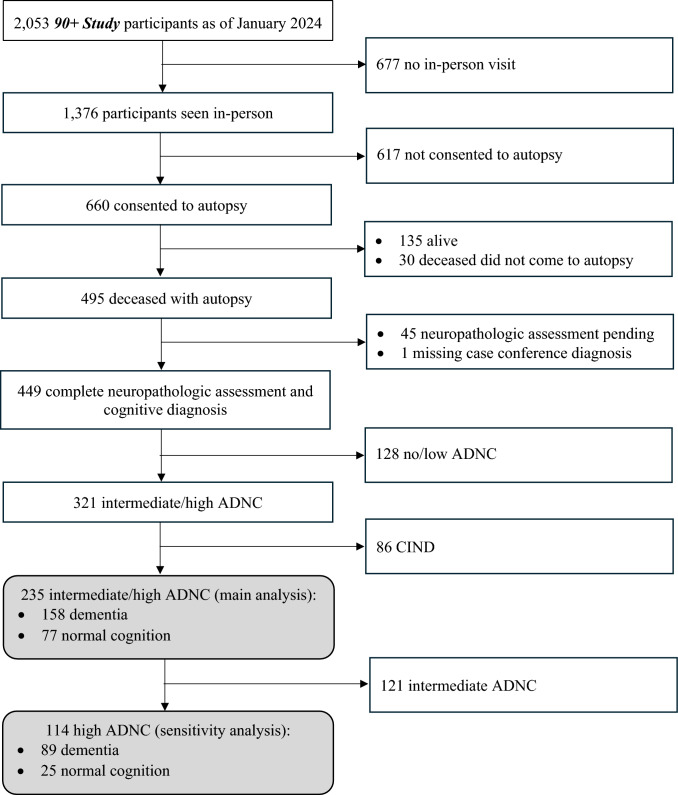
Flowchart of inclusion of participants with intermediate/high ADNC and dementia, and intermediate/high ADNC and normal cognition in the analysis

***The 90* + *Study*** participants with at least one in-person evaluation were invited to participate in the autopsy study. We analyzed data from the subset of autopsied participants who had intermediate or high ADNC at the neuropathological evaluation and normal cognition or dementia as the final cognitive diagnosis assigned at a multidisciplinary consensus case conference after death. Individuals with CIND were excluded because they may represent a prodromal dementia stage. Figure 1 shows the process for selection of participants into this analysis. Of the 2,053 participants enrolled in ***The 90* + *Study***, 1,376 had at least one in-person evaluation and were invited to participate in the autopsy study. Out of those, 660 consented and 449 had died, had autopsy examination completed, and had final cognitive diagnosis assigned. Of those, 235 had intermediate or high ADNC and normal cognition or dementia as a final cognitive diagnosis, and were included in the main analysis. Of those, 114 had high ADNC only and were included in the sensitivity analysis.

### Data analysis

To examine health history and lifestyle factors that distinguish oldest old with dementia from those with normal cognition in the presence of intermediate/high ADNC (Aim 1), we used multiple logistic regression to estimate the odds ratios (OR) and 95% confidence intervals (CI). For all analyses, our outcome of interest was dementia vs. normal cognition, and we modeled the probability of having dementia. Therefore, in our results, an OR above 1 indicates a higher likelihood of dementia. Conversely, an OR below 1 would indicate a higher likelihood of normal cognition. The models were adjusted for continuous age at death, sex, and education (< College degree = reference vs. ≥ College degree) (Model 1). Additionally, we report counts and percentages for categorical variables of demographic characteristics, health history, lifestyle factors, and the mean and standard deviation for the continuous variable of age.

To address potential sources of bias that could affect the interpretation of our primary findings, we conducted two supplementary analyses. First, some health histories and lifestyle factors may be associated with increased mortality risk, which would create a competing risk of dementia for those factors. To account for this, we conducted a survival analysis focused on factors for which the observed direction of the association was contrary to expectation. We used a Cox proportional hazards model that included all the participants of ***The 90* + *Study*** who were seen in person, N = 1,376 to model all-cause mortality with age as the time scale and age at death as the primary outcome. Participants were considered at risk for death and contributed person-years from the age at their first in-person evaluation until either (1) age at death or (2) age at administrative censoring for those still alive. Second, since participants who consented to the autopsy study may differ from those who did not, we examined potential differences in the distribution of health histories and lifestyle factors between these two groups using a chi-squared test.

To examine neuropathological changes that distinguish oldest-old individuals with dementia from those with normal cognition in the presence of intermediate/high ADNC (Aim 2), we used logistic regression to estimate ORs and 95% CIs. The models were adjusted for continuous age at death, sex, and education (< College degree (reference) vs. ≥ College degree) (Model 1). To account for health histories that were associated with higher odds of dementia in the above health and lifestyle factors analysis, we additionally adjusted Model 1 for heart disease, hypertension, and TIA (Model 2). To control for AD severity, we additionally adjusted Model 2 for ADNC score (intermediate vs high) (Model 3). In this last model, all variance inflation factors (VIFs) were below 1.5, indicating no problems with multicollinearity when adjusting for ADNC score. Across all models, Hosmer–Lemeshow goodness-of-fit tests did not indicate a lack of fit (all p > 0.05). We report counts and percentages of each dichotomized neuropathologic change and compare them using chi-squared and Fisher’s exact tests. We also report counts and percentages for the original scoring of each neuropathologic change in Supplementary Table 1. To further account for the effects of potential differences in ADNC severity, we conducted a supplementary analysis by repeating the neuropathological changes analyses above (Models 1 and 2) restricted to participants with high ADNC.

Due to potential sex differences in frequencies of different neuropathologic changes [6] and in the number of individuals with and without dementia in the presence of high levels of neuropathologic change [5], we explored the sex-specific association between each individual neuropathologic change and dementia, vs. normal cognition, in the presence of intermediate/high ADNC using multiple logistic regression analysis stratified by sex, adjusting for age at death and education. This analysis was conducted only for the larger group of participants with intermediate/high ADNC. The number of participants with high ADNC was insufficient for stratified analysis.

Analyses were done using SAS 9.4 (SAS Institute Inc., Cary, NC).

## Results

### Participant characteristics

Among the 235 participants with intermediate/high ADNC, 158 (67%) had dementia and 77 (33%) had normal cognition (Table 1). Average age at death was 98 years for both groups. Women comprised 73% of participants with dementia and 62% of those with normal cognition. College education or higher was found in 41% of participants with dementia and in 55% of those with normal cognition.

**Table 1:** Demographic and health characteristics of participants with intermediate/high and high ADNC by cognitive status, and odds ratios of dementia in relation to demographic and health characteristics in participants with intermediate/high and high ADNC

Characteristics	Intermediate/High ADNC (N = 235)				High ADNC (N = 114)			
	Normal Cognition (*N* = 77)	Dementia (*N* = 158)	OR (95% CI)	*p*-value	Normal cognition (*N* = 25)	Dementia (*N* = 89)	OR (95% CI)	*p*-value
	Mean (SD)							
Age at death, y	97.8 (3.0)	98.0 (3.7)	1.00 (0.93, 1.00)	0.93	98.6 (3.1)	97.8 (3.9)	0.95(0.84, 1.08)	0.45
	*N* (%)							
Sex								
Men	029 (38)	042 (27)	1.00 (reference)	–	7 (28)	22 (25)	1.00 (reference)	–
Women	048 (62)	116 (73)	1.52 (0.83, 2.78)	0.17	18 (72)	67 (75)	0.99 (0.35, 2.81)	0.98
Education								
< College degree	035 (45)	093 (59)	1.00 (reference)	–	07 (28)	50 (56)	1.00 (reference)	–
≥ College degree	042 (55)	065 (41)	0.62 (0.36, 1.09)	0.10	18 (72)	39 (44)	0.31 (0.12, 0.83)	**0.02**
APOE status								
ε2	015 (21)	022 (15)	0.68 (0.32, 1.43)	0.30	4 (17)	10 (13)	0.59 (0.15, 2.27)	0.44
ε4	016 (22)	045 (31)	1.60 (0.82, 3.11)	0.17	8 (33)	21 (26)	0.74 (0.27, 2.04)	0.56
Cardiovascular disease history								
Diabetes	008 (10)	009 (6)	0.56 (0.20, 1.55)	0.26	2 (8)	4 (5)	0.37 (0.06, 2.40)	0.30
Heart disease^a^	052 (68)	073 (47)	0.45 (0.25, 0.81)	** < 0.01**	17 (68)	39 (44)	0.45 (0.17, 1.21)	0.11
Hypercholesterolemia	031 (41)	042 (28)	0.56 (0.31, 1.00)	0.05	11 (46)	24 (29)	0.463 0.18 1.23	0.12
Hypertension	050 (65)	081 (51)	0.53 (0.29, 0.95)	**0.03**	15 (60)	41 (48)	0.48 (0.18, 1.28)	0.14
Neurological disease history								
Head trauma	006 (8)	021 (13)	1.64 (0.63, 4.31)	0.31	3 (12)	9 (10)	0.73 (0.17, 3.14)	0.67
Parkinson’s disease	001 (1)	005 (3)	2.91 (0.33, 25.97)	0.34	1 (4)	3 (3)	0.98 (0.09, 10.48)	0.99
Seizures	0	006 (4)	NC^b^	NC	0	2 (2)	NC	NC
Stroke	005 (7)	024 (16)	2.69 (0.97, 7.45)	0.06	1 (4)	9 (10)	3.01 (0.34, 26.70)	0.32
TIA	012 (16)	051 (34)	3.00 (1.46, 6.18)	** < 0.01**	3 (12)	28 (33)	4.36 (1.16, 16.41)	**0.03**
Other disease history								
Cancer^c^	44 (64)	77 (61)	0.88 (0.47, 1.65)	0.70	12 (60)	49 (65)	1.27 (0.43, 3.73)	0.66
Cataracts	065 (84)	115 (75)	0.57 (0.27, 1.17)	0.12	20 (80)	63 (74)	0.83 (0.27, 2.59)	0.75
Rheumatoid arthritis	009 (12)	009 (6)	0.45 (0.17,1.22)	0.12	3 (13)	4 (5)	0.32 (0.06, 1.78)	0.19
Thyroid disease	022 (29)	048 (32)	1.04 (0.55, 1.94)	0.91	8 (32)	24 (28)	0.85 (0.31, 2.36)	0.76
Body mass index	24.1 (4)	24.8 (5)	1.06 (0.97, 1.14)	0.19	23.8 (4)	24.1 (5)	1.03 (0.90, 1.17)	0.68
Lifestyle factors								
Smoking^d^	36 (47)	70 (45)	0.98 (0.56, 1.72)	0.95	12 (48)	36 (42)	0.86 (0.34, 2.18)	0.76
Alcohol	51 (67)	92 (59)	0.74 (0.41, 1.33)	0.32	16 (64)	55 (63)	1.02 (0.38, 2.72)	0.97

Number of participants with intermediate/high ADNC missing: APOE = 16, diabetes = 2, heart disease = 2, hypercholesterolemia = 11, hypertension = 4, head trauma = 5, Parkinson’s disease = 3, seizures = 4, stroke = 4, TIA = 9, cataracts = 5, rheumatoid arthritis = 6, thyroid disease = 7, cancer = 39, smoking = 5, BMI = 70, drinking = 3

Number of participants with High ADNC missing: APOE = 10, diabetes = 1, heart disease = 1, hypercholesterolemia = 7, hypertension = 3, head trauma = 2, Parkinson’s disease = 1, seizures = 3, stroke = 2, TIA = 4, cataracts = 4, rheumatoid arthritis = 3, thyroid disease = 4, cancer = 19, smoking = 3, BMI = 40, drinking = 2

Odds Ratios (OR) and their 95% Confidence Intervals (CI) from logistic regression with binary health characteristics as predictors (absence of condition = reference) and dementia vs. normal cognition as the outcome, adjusted for age at death, sex, and education. Significant findings (*p* < 0.05) are in bold

^a^Heart disease includes any of the following: coronary artery disease, myocardial infarction, arrhythmia, heart valve disease, congestive heart failure, coronary artery bypass, and pacemaker

^b^NC = Not calculated due to sparse cells

^c^Cancer includes any of the following: melanoma, other skin cancer, colon, uterine, breast, prostate, and other cancers; smoking = ever smoking; body mass index = ; alcohol = alcohol consumption ever

ADNC = Alzheimer’s disease neuropathologic change; TIA = transient ischemic attack

### Health history and lifestyle factors in relation to dementia vs. normal cognition

We used multiple logistic regression adjusted for age at death, sex, and education to examine the association between health history and lifestyle factors and the odds of having dementia vs. normal cognition. Participants with heart disease (OR = 0.45; 95% CI = 0.25, 0.81) or with hypertension (OR = 0.53; 95% CI = 0.29, 0.95) were less likely to have dementia (Table 1). In contrast, participants with transient ischemic attacks (TIAs) were more likely to have dementia (OR = 3.00; 95%CI = 1.46, 6.18).

We conducted a survival analysis to examine the competing risk of death in participants with and without certain health histories and lifestyle factors for which the direction of the association with dementia was contrary to expectation. Risk of death was increased in participants with diabetes (HR = 1.27; 95%CI = 1.04, 1.55) and heart disease (HR = 1.30; 95%CI = 1.16, 1.46). Conversely risk of death was decreased in participants with hypercholesterolemia (HR = 0.88; 95%CI = 0.77, 0.99), cataracts (HR = 0.80; 95%CI = 0.69, 0.93), and a history of alcohol use (HR = 0.80; 95%CI = 0.72, 0.91). No significant association with mortality was observed for hypertension (Table 2).

**Table 2:** Association between selected health histories and mortality among all participants of *The 90* + *Study* seen in person (*N* = 1376)

Health histories	N	HR (95% CI)	*p*-value
Cardiovascular disease			
Diabetes	1342	1.27 (1.04, 1.55)	**0.02**
Heart disease	1348	1.30 (1.16, 1.46)	** < 0.01**
Hypercholesterolemia	1277	0.88 (0.77, 0.99)	**0.04**
Hypertension	1325	1.03 (0.91, 1.15)	0.68
Other disease			
Cancer	963	1.00 (0.87, 1.16)	0.96
Cataracts	1284	0.80 (0.69, 0.93)	** < 0.01**
Rheumatoid arthritis	1318	1.13 (0.90, 1.41)	0.31
Lifestyle factors			
Smoking	1319	1.07 (0.95, 1.20)	0.25
Alcohol	1289	0.80 (0.72, 0.91)	** < 0.01**

*N* number of participants included in the analysis. *N* differs by history due to missing data

*HR* hazard ratio, *CI* confidence interval

HR, 95%CI, and *p*-values from survival analysis of participants with and without health histories associated with dementia, using Cox proportional hazard model

We used a chi-squared test to examine differences in the distributions of health histories and lifestyle factors between participants who did and did not consent to the autopsy study. Compared to those who did not consent, participants who consented more frequently had hypercholesterolemia (39% vs. 30%, *p* < 0.01) and a history of any cancer (60% vs. 68%, *p* = 0.01), and reported less frequent alcohol use (54% vs. 62%, *p* < 0.01) (Table 3).

**Table 3:** Comparison of selected health histories in people consenting to autopsy and those who did not among all participants of *The 90* + *Study* seen in person (N = 1376)

Health histories	Consented to autopsy		Total (*N* = 1376)	*p*-value
	Yes (*N* = 660)	No (*N* = 716)		
	*N* (%)			
Cardiovascular disease				
Diabetes	61 (10)	66 (9)	127 (9)	0.95
Heart disease^a^	303 (47)	323 (46)	626 (46)	0.81
Hypercholesterolemia	243 (39)	195 (30)	438 (34)	** < 0.01**
Hypertension	361 (56)	373 (54)	734 (55)	0.36
Other disease				
Cancer^b^	319 (60)	296 (68)	615 (63)	**0.01**
Cataracts	515 (82)	516 (78)	1031 (80)	0.09
Rheumatoid arthritis	38 (6)	50 (7)	88 (7)	0.36
Lifestyle factors				
Smoking	299 (47)	308 (45)	607 (46)	0.56
Alcohol	391 (62)	361 (54)	752 (58)	** < 0.01**

*p*-values from chi-squared test

^a^Heart disease includes any of the following: coronary artery disease, myocardial infarction, arrhythmia, heart valve disease, congestive heart failure, coronary artery bypass, and pacemaker

^b^Cancer includes any of the following: melanoma, other skin cancer, colon, uterine, breast, prostate, and other cancers

### Distribution of individual neuropathologic changes in participants with dementia vs. normal cognition

The distribution of dichotomized neuropathologic changes by dementia vs. normal cognition is shown in Table 4 (Supplementary Table 1 shows distribution using the original detailed categorization). High levels of neurodegenerative neuropathologic changes were more frequent in participants with dementia, compared with those with normal cognition: high ADNC level 56% vs. 33%, Thal phase 4/5 77% vs. 61%, tangle Braak stage V/VI 64% vs. 36%, LBD limbic/cortical 21% vs. 9%, HS 20% vs. 8%, LATE-NC 51% vs. 29%, a higher number (3 +) of non-ADNCs 51% vs. 26%. High levels of vascular neuropathologic changes were distributed relatively equally between participants with dementia and participants with normal cognition: arteriolosclerosis 86% vs. 87%, severe atherosclerosis 8% vs. 3%, CAA 69% vs. 64%, three or more MVLs 6% vs. 1%.

**Table 4:** Frequencies of dichotomized neuropathologic changes for intermediate/high ADNC and high ADNC by cognitive status

	Intermediate/high ADNC (*N* = 235)		High ADNC (*N* = 114)	
	Normal cognition (*N* = 77)	Dementia (*N* = 158)	Normal cognition (*N* = 25)	Dementia (*N* = 89)
	*N* (%)			
ADNC				
Intermediate	052 (68)	069 (44)	0	0
High	025 (32)	089 (56)	25 (100)	89 (100)
Thal phase				
1/2/3	030 (39)	037 (23)	0	0
4/5	047 (61)	121 (77)	25 (100)	89 (100)
Tangle Braak stage				
III/IV	049 (64)	057 (36)	0	0
V/VI	028 (36)	101 (64)	25 (100)	89 (100)
CERAD score				
None/sparse/moderate	24 (31)	38 (24)	00	02 (2)
Frequent	53 (69)	120 (76)	25 (100)	87 (98)
LBD				
None/olfactory	070 (91)	125 (79)	22 (88)	69 (78)
Limbic/cortical	007 (9)	033 (21)	03 (12)	20 (22)
HS				
No	071 (92)	127 (80)	24 (96)	71 (80)
Yes	006 (8)	031 (20)	01 (4)	18 (20)
LATE-NC				
None	055 (71)	078 (49)	19 (76)	40 (45)
Amygdala/hippocampus/cortex	022 (29)	080 (51)	06 (24)	49 (55)
Arteriolosclerosis				
None	010 (13)	022 (14)	01 (4)	13 (15)
Mild/moderate/severe	067 (87)	136 (86)	24 (96)	76 (85)
Arteriolosclerosis				
None/mild	30 (39)	64 (41)	5 (20)	39 (44)
Moderate/severe	47 (61)	94 (59)	20 (80)	50 (56)
Atherosclerosis^a^				
None/mild/moderate	75 (97)	142 (92)	24 (96)	80 (92)
Severe	2 (3)	13 (8)	01 (4)	07 (8)
Atherosclerosis^a^				
None/mild	50 (65)	94 (61)	53 (61)	16 (64)
Moderate/severe	27 (35)	61 (39)	34 (39)	09 (36)
CAA				
None	028 (36)	049 (31)	07 (28)	21 (24)
Mild/moderate/severe	049 (64)	109 (69)	18 (72)	68 (76)
MVL				
0–1	73 (95)	142 (90)	25 (100)	81 (91)
2 +	4 (5)	16 (10)	00	08 (9)
MVL				
0–2	76 (99)	149 (94)	25 (100)	85 (96)
3 +	1 (1)	9 (6)	00	04 (5)
Number of non-ADNCs				
0–1	022 (29)	021 (13)	6 (24)	11 (12)
2	035 (45)	057 (36)	10 (40)	29 (33)
3–6	020 (26)	080 (51)	9 (36)	49 (55)

*p*-values from chi-squared and Fisher’s exact tests comparing frequencies of neuropathologic changes in dementia vs. normal cognition. Significant findings (*p* < 0.05) are in bold

*ADNC* Alzheimer’s disease neuropathologic change, *CERAD* Consortium to Establish a Registry for Alzheimer’s Disease, *LBD* Lewy bodies disease, *HS* Hippocampal sclerosis, *LATE-NC* limbic predominant age-related TDP-43 encephalopathy neuropathologic change, *CAA* Cerebral amyloid angiopathy, *MVL* microvascular lesions

^a^Three participants missing atherosclerosis data

### Individual neuropathologic changes in relation to dementia vs. normal cognition

We used multiple logistic regression adjusted for age at death, sex, and education to examine the association between each individual neuropathologic change and the odds of having dementia vs. normal cognition. Participants with a high AD score (OR = 2.83; 95% CI = 1.57, 5.09), Thal phase (4/5) (OR = 2.24; 95% CI = 1.22, 4.11), tangle Braak stage (V/VI) (OR = 3.16; 95% CI = 1.75, 5.65), LBD (limbic/cortical) (OR = 2.73; 95% CI = 1.14, 6.58), HS (OR = 2.70; 95% CI = 1.06, 6.86), LATE-NC (amygdala, hippocampus, cortex) (OR = 2.80; 95% CI = 1.53, 5.12), and number of non-ADNCs (3 +) (OR = 4.46; 95% CI = 2.01, 9.92) were more likely to have dementia, compared with participants with no or low levels of these neuropathologic changes (Table 5 Model 1). Additional adjustment for health factors associated with higher odds of having dementia (heart disease, hypertension, and TIA) (Table 5 Model 2), and additional adjustment for ADNC (Table 5 Model 3) yielded similar results.

**Table 5:** Odds ratios of having dementia, vs. normal cognition, in participants with intermediate/high or high ADNC in relation to individual dichotomized neuropathologic changes

	Intermediate/High ADNC (*N* = 235)						High ADNC (*N* = 114)			
	Model 1		Model 2		Model 3		Model 1		Model 2	
	OR (95% Cl)	*p*-value	OR (95% Cl)	*p*-value	OR (95% Cl)	*p*-value	OR (95% Cl)	*p*-value	OR (95% Cl)	*p*-value
ADNC										
Intermediate	1.00 (reference)	–	1.00 (reference)	–	NA^a^	NA	NA	NA	NA	NA
High	2.83 (1.57, 5.09)	** < 0.01**	2.55 (1.36, 4.78)	** < 0.01**	NA	NA	NA	NA	NA	NA
Thal phase										
1–3	1.00 (reference)	–	1.00 (reference)	–	NA	NA	NA	NA	NA	NA
4/5	2.24 (1.22, 4.11)	** < 0.01**	2.23 (1.15, 4.30)	**0.02**	NA	NA	NA	NA	NA	NA
Tangle Braak stage										
III/IV	1.00 (reference)	–	1.00 (reference)	–	NA	NA	NA	NA	NA	NA
V/VI	3.16 (1.77, 5.65)	** < 0.01**	2.88 (1.54, 5.37)	** < 0.01**	NA	NA	NA	NA	NA	NA
CERAD score										
None/sparse/mod	1.00 (reference)	–	1.00 (reference)	–	NA	NA	NA	NA	NA	NA
Severe	1.37 (0.74, 2.55)	0.32	1.16 (0.59, 2.28)	0.66	NA	NA	NA	NA	NA	NA
LBD										
None/olfactory	1.00 (reference)	–	1.00 (reference)	–	1.00 (reference)	–	1.00 (reference)	–		
Limbic/cortical	2.73 (1.14, 6.58)	**0.02**	2.93 (1.16, 7.45)	**0.02**	2.86 (1.09, 7.50)	**0.03**	2.51 (0.65, 9.63)	0.18	2.38 (0.58, 9.76)	0.23
HS										
No	1.00 (reference)	–	1.00 (reference)	–	1.00 (reference)	–	1.00 (reference)	–		
Yes	2.70 (1.06, 6.86)	**0.04**	3.33 (1.23, 9.02)	**0.02**	3.50 (1.27, 9.64)	**0.02**	7.06 (0.85, 58.45)	0.07	13.43 (1.28, 140.57)	**0.03**
LATE-NC										
None	1.00 (reference)	–	1.00 (reference)	–	1.00 (reference)	–	1.00 (reference)	–		
Amyg/hippoc/cortex	2.80 (1.53, 5.12)	** < 0.01**	2.96 (1.53, 5.71)	** < 0.01**	2.98 (1.52, 5.83)	** < 0.01**	6.21 (2.03, 18.98)	** < 0.01**	9.85 (2.49, 38.94)	** < 0.01**
Arteriolosclerosis										
None	1.00 (reference)	–	1.00 (reference)	–	1.00 (reference)	–	1.00 (reference)	–	1.00 (reference)	–
Mild/mod/severe	0.90 (0.40, 2.04)	0.80	0.95 (0.39, 2.29)	0.91	0.90 (0.36, 2.25)	0.82	0.29 (0.03, 2.50)	0.26	0.29 (0.03, 2.66)	0.26
Arteriolosclerosis										
none/mild	1.00 (reference)	–	1.00 (reference)	–	1.00 (reference)	–	1.00 (reference)	–	1.00 (reference)	–
Moderate/severe	0.88 (0.50, 1.57)	0.68	0.79 (0.42, 1.46)	0.45	0.74 (0.39, 1.41)	0.36	0.34 (0.11, 1.04)	0.06	0.33 (0.101 1.039	0.06
Atherosclerosis^c^										
None/mild/mod	1.00 (reference)	–	1.00 (reference)	–	1.00 (reference)	–	1.00 (reference)	–	1.00 (reference)	–
Severe	3.49 (0.76, 16.15)	0.11	2.12 (0.42, 10.71)	0.36	1.96 (0.39, 9.84)	0.42	1.95 (0.22, 17.55)	0.55	1.44 (0.14, 15.22)	0.76
Atherosclerosis										
None/mild	1.00 (reference)	–	1.00 (reference)	–	1.00 (reference)	–	1.00 (reference)	–	1.00 (reference)	–
Mod/severe	1.21 (0.68, 2.2)	0.51	1.18 (0.63, 2.21)	0.60	1.15 (0.61, 2.17)	0.66	1.30 (0.50, 3.38)	0.59	1.76 (0.58, 5.35)	0.32
CAA										
None	1.00 (reference)	–	1.00 (reference)	–	1.00 (reference)	–	1.00 (reference)	–	1.00 (reference)	–
Mild/mod/severe	1.30 (0.72, 2.32)	0.38	1.16 (0.61, 2.18)	0.65	1.03 (0.54, 1.98)	0.92	1.25 (0.44, 3.53)	0.68	1.05 (0.34, 3.26)	0.93
MVL										
0–1	1.00 (reference)	–	1.00 (reference)	–	1.00 (reference)	–	NC^b^	NC	NC	NC
2 +	2.00 (0.63, 6.30)	0.24	1.27 (0.35, 4.68)	0.72	1.60 (0.42, 6.15)	0.50	NC	NC	NC	NC
MVL										
0–2	1.00 (reference)	–	1.00 (reference)	–	1.00 (reference)	–	NC	NC	NC	NC
3 +	5.08 (0.62, 41.50)	0.13	2.94 (0.29, 30.28)	0.37	3.71 (0.34, 41.09)	0.29	NC	NC	NC	NC
N non–ADNCs										
0–1	1.00 (reference)	–	1.00 (reference)	–	1.00 (reference)	–	1.00 (reference)	–	1.00 (reference)	–
2	1.68 (0.79, 3.60)	0.18	1.38 (0.60, 3.19)	0.45	1.45 (0.62, 3.41)	0.40	1.63 (0.43, 6.20)	0.47	1.24 (0.29, 5.31)	0.77
3–6	4.46 (2.01, 9.92)	** < 0.01**	4.27 (1.78, 10.23)	** < 0.01**	4.04 (1.66, 9.85)	** < 0.01**	4.11 (1.06, 15.92)	**0.04**	4.59 (1.00, 21.01)	**0.049**

Odds ratios (OR) and their 95% Confidence intervals (CI) from logistic regression with dichotomized neuropathologic change as predictor (absence or lowest levels of neuropathologic change = reference), and dementia vs. normal cognition as the outcome, adjusted for age at death, sex, and education (model 1), additionally adjusted for heart disease, hypertension, and TIA (model 2); Model 2 + ADNC (model 3). Significant findings (*p* < 0.05) are in bold

*ADNC* Alzheimer’s disease neuropathologic change, *CERAD* Consortium to Establish a Registry for Alzheimer’s Disease, *LBD* Lewy bodies disease, *HS* Hippocampal sclerosis, *LATE-NC* limbic predominant age-related TDP-43 encephalopathy neuropathologic change, *CAA* cerebral amyloid angiopathy, *MVL* microvascular lesions

^a^*NA* not calculated because part of ADNC definition

^b^*NC* not calculated due to sparse cells

^c^Three participants miss atherosclerosis data

### Individual neuropathologic changes in relation to dementia vs. normal cognition by sex

We used multiple logistic regression analysis stratified by sex and adjusted for age at death and education to examine the sex-specific association between each individual neuropathologic change and the odds of dementia vs. normal cognition. We found that women with a high AD score (OR = 2.53; 95% CI = 1.24, 5.15), tangle Braak stage (V/VI) (OR = 3.37; 95% CI = 1.65, 6.88), LATE-NC (amygdala, hippocampus, cortex) (OR = 2.73; 95% CI = 1.28, 5.80), and a number of non-ADNCs (3 +) (OR = 5.89; 95% CI = 2.24, 15.50) were more likely to have dementia, compared with women with no or low levels of these neuropathologic changes (Table 6). Men with a high AD score (OR = 5.05; 95% CI = 1.56, 16.36), Thal phase (4/5) (OR = 3.64; 95% CI = 1.21, 10.97), tangle Braak stage (V/VI) (OR = 3.51; 95% CI = 1.19, 10.30), and LATE-NC (amygdala, hippocampus, cortex) (OR = 2.80; 95% CI = 1.01, 7.81) were more likely to have dementia compared with men with no or low levels of these neuropathologic changes.

**Table 6:** Odds ratios of having dementia vs normal cognition in relation to individual neuropathologic changes for men and women

	Women (*N* = 164)				Men (*N* = 71)			
	Normal cognition (*N* = 48)	Dementia (*N* = 116)	OR (95% CI)	*p*-value	Normal cognition (*N* = 29)	Dementia (N = 42)	OR (95% CI)	*p*-value
	N (%)							
ADNC								
Intermediate	030 (63)	049 (42)	1.00 (reference)	–	22 (76)	20 (48)	1.00 (reference)	–
Severe	018 (37)	067 (58)	2.53 (1.24, 5.15)	**0.01**	7 (24)	22 (52)	5.05 (1.56, 16.36)	**0.01**
Thal phase								
1–3	017 (35)	028 (24)	1.00 (reference)	–	13 (45)	9 (21)	1.00 (reference)	–
4/5	031 (65)	088 (76)	1.94 (0.91, 4.14)	0.08	16 (55)	33 (79)	3.64 (1.21, 10.97)	**0.02**
Tangle Braak stage								
III/IV	029 (60)	038 (33)	1.00 (reference)	–	20 (69)	19 (45)	1.00 (reference)	–
V/VI	019 (40)	078 (67)	3.37 (1.65, 6.88)	** < 0.01**	9 (31)	23 (55)	3.51 (1.19, 10.30)	**0.02**
CERAD score								
None/sparse/moderate	012 (25)	025 (22)	1.00 (reference)	–	12 (41)	13 (31)	1.00 (reference)	–
Severe	036 (75)	091 (78)	1.28 (0.57, 2.85)	0.55	17 (59)	29 (69)	1.78 (0.64, 4.92)	0.27
LBD								
None/olfactory	043 (90)	091 (79)	1.00 (reference)	–	27 (93)	34 (81)	1.00 (reference)	–
Limbic/neocortical	005 (10)	025 (21)	2.66 (0.94, 7.57)	0.07	2 (7)	08 (19)	3.28 (0.63, 16.99)	0.15
HS								
No	043 (90)	090 (78)	1.00 (reference)	–	28 (97)	37 (88)	1.00 (reference)	–
Yes	005 (10)	026 (22)	2.44 (0.87, 6.85)	0.09	1 (3)	5 (12)	3.89 (0.42, 36.17)	0.23
LATE-NC								
None	034 (71)	058 (50)	1.00 (reference)	–	21 (72)	20 (48)	1.00 (reference)	–
Amygdala/hippocampus/cortex	014 (29)	058 (50)	2.73 (1.28, 5.80)	**0.01**	8 (28)	22 (52)	2.80 (1.01, 7.81)	**0.05**
Arteriolosclerosis								
None	007 (15)	015 (13)	1.00 (reference)	–	3 (10)	7 (17)	1.00 (reference)	–
Mild/moderate/severe	041 (85)	101 (87)	1.19 (0.45, 3.16)	0.72	26 (90)	35 (83)	0.58 (0.13, 2.62)	0.48
Arteriolosclerosis								
None/mild	18 (38)	44 (38)	1.00 (reference)	–	12 (41)	20 (48)	1.00 (reference)	–
Moderate/severe	30 (62)	72 (62)	0.94 (0.46, 1.90)	0.86	17 (59)	22 (52)	0.85 (0.32, 2.28)	0.75
Atherosclerosis^a^								
None/mild/moderate	048 (100)	104 (92)	1.00 (reference)	–	27 (93)	38 (91)	1.00 (reference)	–
Severe	000 (0)	009 (8)	NC^b^	NC	2 (7)	04 (9)	1.28 (0.21, 7.72)	0.79
Atherosclerosis								
None/mild	032 (67)	070 (62)	1.00 (reference)	–	18 (62)	24 (57)	1.00 (reference)	–
Moderate/severe	016 (33)	043 (38)	1.19 (0.58, 2.44)	0.63	11 (38)	18 (43)	1.19 (0.45, 3.18)	0.72
CAA								
None	018 (38)	034 (29)	1.00 (reference)	–	10 (34)	15 (36)	1.00 (reference)	–
Mild/moderate/severe	030 (62)	082 (71)	1.56 (0.76, 3.21)	0.23	19 (66)	27 (64)	1.01 (0.37, 2.78)	0.99
MVL								
0–1	046 (96)	102 (88)	1.00 (reference)	–	27 (93)	40 (95)	1.00 (reference)	–
2 +	002 (4)	014 (12)	3.05 (0.66, 14.12)	0.15	2 (7)	2 (5)	0.80 (0.10, 6.24)	0.83
MVL								
0–1	048 (100)	109 (94)	1.00 (reference)	–	28 (97)	40 (95)	1.00 (reference)	–
3 +	000 (0)	007 (6)	NC	NC	1 (3)	2 (5)	1.75 (0.14, 21.44)	0.67
Non-ADNC								
0–1	016 (33)	014 (12)	1.00 (reference)	–	6 (21)	7 (17)	1.00 (reference)	–
2	019 (40)	043 (37)	2.45 (0.98, 6.11)	0.06	16 (55)	14 (33)	0.87 (0.21, 3.53)	0.84
3–6	013 (27)	059 (51)	5.89 (2.24, 15.50)	** < 0.01**	7 (24)	21 (50)	2.93 (0.67, 12.86)	0.16

Odds ratios (OR) and their 95% confidence intervals (CI) from logistic regression with dichotomized neuropathologic change as predictor (absence or lowest levels of neuropathologic change = reference), and dementia vs. normal cognition as the outcome, adjusted for age at death and education. Significant findings (*p* < 0.05) are in bold

*ADNC* Alzheimer’s disease neuropathologic change, *CERAD* Consortium to Establish a Registry for Alzheimer’s Disease, *LBD* Lewy bodies disease, *HS* Hippocampal sclerosis, *LATE-NC* limbic predominant age-related TDP-43 encephalopathy neuropathologic change, *CAA* cerebral amyloid angiopathy, *MVL* microvascular lesions

^a^Three participants miss atherosclerosis data

^b^*NC* not calculated due to sparse cells

### Sensitivity analysis

We conducted sensitivity analysis by repeating the above analyses for participants with high ADNC only to account for the effects of potential differences in ADNC severity. Of the 114 participants with high ADNC, 89 (78%) had dementia, and 25 (22%) had normal cognition (Table 1). The average age at death was 98 years for participants with dementia, and 99 years for those with normal cognition. Women comprised 75% of participants with dementia and 72% of participants with normal cognition. A college education or higher was reported in 44% of participants with dementia and in 72% of participants with normal cognition.

Participants with college education or higher (OR = 0.31; 95% CI = 0.12, 0.83) were less likely to have dementia, compared with participants with below college education (Table 1). Similarly to the main analysis, participants with transient ischemic attacks (TIAs) were more likely to have dementia (OR = 4.36; 95% CI = 1.16, 16.41), compared with participants without TIAs.

The distribution of dichotomized neuropathologic changes in participants with dementia and normal cognition is presented in Table 4 (Supplementary Table 1 shows the distribution using the original categories). High levels of neurodegenerative neuropathologic changes were seen more frequently in participants with dementia, compared with participants with normal cognition: LBD limbic/cortical 22% vs. 12%, HS 20% vs. 4%, LATE-NC 55% vs. 24%. High levels of vascular neuropathologic changes were distributed relatively equally between participants with dementia and participants with normal cognition: arteriolosclerosis 85% vs. 96%, severe atherosclerosis 8% vs. 4%, CAA 76% vs. 72%, three or more MVLs 5% vs. 0%.

Similarly to the main analysis, participants with the presence of LATE-NC (OR = 6.21; 95%CI = 2.03, 18.98) and a higher number (3 +) of non-ADNCs (OR = 4.11; 95%CI = 1.06, 15.92) were more likely to have dementia, compared with participants with no or low levels of these neuropathologic changes (Table 5).

## Discussion

This study aimed to extend previous research by providing additional evidence for health, lifestyle, and neuropathologic factors that distinguish older adults with dementia vs. normal cognition in the presence of intermediate or high ADNC. We found that among participants with intermediate/high ADNC, 67% had dementia and 33% had normal cognition, and among participants with high ADNC, 78% had dementia and 22% had normal cognition. Participants with a college education or higher, heart disease, and hypertension were more likely to be in the normal cognition group, whereas participants with transient ischemic attacks, high AD score, Thal phase, tangle Braak stage, presence of LBD, HS, LATE-NC, and a higher number of non-ADNCs were more likely to be in the dementia group. Looking at the distribution of neuropathologic changes between the oldest old with dementia and those with normal cognition, we found that neurodegenerative neuropathologic changes were more frequent in the dementia group, whereas vascular neuropathologic changes were distributed relatively equally between the dementia and normal cognition groups.

In this oldest old cohort, 22–33% of participants with intermediate or high ADNC remained cognitively normal until death. These numbers agree with other studies that report 24–28% of older adults having normal cognition and moderate/severe ADNC regardless of other neuropathologic changes [7, 30]. One study that has done an analysis similar to ours and has used a more restrictive definition of resilience—normal cognition, severe ADNC, and no other primary neuropathology—understandably reports a much lower prevalence of 9% [1].

Our results show that participants with a college education or higher were less likely to have dementia, i.e., more likely to have normal cognition. This result agrees with a similar study where NACC participants with normal cognition in the presence of severe NIA-Reagan ADNC had higher education compared to those with dementia [1]. Our result is also consistent with studies that compared older adults with and without dementia, finding that lower likelihood of dementia was associated with higher education [2, 23]. Our result is also consistent with the notion of cognitive reserve, where education allows for more efficient use of brain networks, which protects against cognitive impairment in the face of high levels of neuropathologic change including the ADNC [18]. Additionally, higher education may increase the ability to perform on tests, thus hiding cognitive impairment.

We found that heart disease and hypertension were associated with a lower likelihood of having dementia, whereas TIAs were associated with a higher likelihood of dementia. Survival analysis showed that participants with heart disease, compared with those without heart disease, had a higher mortality risk, and therefore they may not survive long enough to develop dementia. This selective survival can produce a seemingly “protective” association between heart disease and dementia in the current analyses. Thus, the inverse association observed in this study is likely attributable, at least in part, to survivor bias rather than a true protective effect of heart disease on cognitive outcomes. However, our finding of a lower risk of dementia in individuals with hypertension is consistent with some previous reports [39], but not others [2]. Specifically, in NACC autopsy participants with an average age at death of 85 years, those with no dementia vs. dementia, and substantial ADNC (moderate/ frequent CERAD and V/ VI Braak stages) more frequently had cardiovascular risk factors and heart disease (hypertension, heart failure, and atrial fibrillation) [39]. One explanation for our findings could be that medications taken for hypertension provide some cognitive protection, for example through an anti-inflammatory effect that interferes with the chronic inflammation commencing before dementia onset and continuing throughout its course, thus protecting against clinical onset of AD [4, 24, 40]. Another explanation could be that hypertension in late life protects against dementia by maintaining adequate cerebral perfusion in the presence of age-related vascular changes. In support of this hypothesis, low cerebral blood flow has been associated with higher rates of cognitive decline and prevalent dementia [10, 19, 36]. By contrast, cerebrovascular disease (TIAs) results in direct brain damage thus affecting cognitive functioning.

In this study, a high ADNC score, the Thal phase, the tangle Braak stage, the presence of LBD, LATE-NC, HS, and a high number of non-ADNCs were associated with a higher likelihood of dementia. Of note, the strength of the association of Thal phase with the likelihood of dementia was somewhat smaller than that of non-ADNCs, which is consistent with the understanding that amyloid becomes a weaker determinant for dementia with advancing age [37]. Our finding that multiple neurodegenerative neuropathologic changes are associated with dementia in the oldest old with intermediate/high ADNC is consistent with a similar study that showed that NACC autopsy participants with normal cognition and moderate/high NIA-AA ADNC had lower severity of LATE-NC and LBD and lower likelihood of arteriosclerosis compared to those with dementia [12]. Research comparing older adults with and without dementia also supports the idea that multiple neuropathologic changes have an interactive effect on the risk of dementia, including LATE-NC, LBD, HS, and microinfarcts [2]. In our study, vascular neuropathologic changes in the presence of ADNC were not related to the odds of dementia, which is in line with one [1], but not with the other [12] similar prior studies. Our finding is consistent with the understanding that vascular neuropathologic changes might contribute less to cognitive impairment than neurodegenerative neuropathologic changes [13, 25]. Taken together, these findings support the idea that dementia in the presence of intermediate/high ADNC may, at least in part, depend on the additional impact of non-ADNCs. Additional neuropathologic changes may reduce the brain’s ability to withstand damage and are associated with cognitive impairment and dementia [11, 15, 16]. Additionally, we found that high levels of neurodegenerative neuropathologic changes (ADNC, Thal phase 4/5, tangle Braak stage V/VI, LBD limbic/cortical, HS, LATE-NC, higher number of non-ADNCs) were observed more frequently in participants with dementia, whereas higher levels of vascular neuropathologic changes (arteriolosclerosis, severe atherosclerosis, CAA, three or more MVLs) were distributed similarly between those with dementia and those with normal cognition. This is in line with a previous report where participants of the ACT study with Braak VI, CERAD frequent and dementia had a higher burden of phosphorylated tau, amyloid plaques, LATE-NC, and macroscopic infarcts, compared with those without dementia [20].

Finally, in the sex-specific analysis, higher likelihood of dementia was associated, in women, with high AD score, tangle Braak stage, LATE-NC, and higher number of non-ADNCs, and in men, it was associated with high AD score, Thal phase, tangle Braak stage, and LATE-NC. In other words, multiple non-ADNCs are present in women with dementia, whereas high ADNCs are present in men with dementia. Though no consensus in the field exists [5], several studies have demonstrated more resilience and faster adaptation to tau neuropathologic change in older women than men [22, 42]. A recent review on sex differences in cognitive resilience indicated that women show greater cognitive resilience than men at initial stages of disease (cross-sectional analysis) with more rapid subsequent decline over time [5]. Additionally, a higher education level in men, compared with women, may contribute to their resilience to non-ADNCs.

### Strengths and limitations

This study has a number of strengths. First, we analyzed data from one of the largest autopsy cohorts of the oldest old. The relatively large sample size allowed for adequate numbers of participants with dementia and normal cognition who harbor intermediate/high ADNC, even when restricting analysis to participants with high ADNC only. Second, ***The 90* + *Study*** cohort is well-characterized using comprehensive cognitive and neurological evaluation. Third, active steps were taken to minimize missing data, which may be high in this age group due to prevalent frailty and sensory-motor impairments. Fourth, individuals age 90 and older allow examination of neuropathologic changes like LATE-NC, HS, vascular, which become more prevalent with increasing age.

We acknowledge several limitations. First, given the number of statistical tests performed, the findings should be interpreted with caution. The reported associations are modest, potentially due to small sample sizes, and might require confirmation using a larger cohort. Our sex-stratified analysis, which yielded relatively wide confidence intervals, was also likely limited in statistical power due to small subgroup sizes. However, the alignment of our findings with previous reports suggests the robustness of our results. The oldest old participants may be different from younger cohorts of older adults in that they are resilient to some age-related factors associated with longevity. Therefore, replication of these findings in younger cohorts might be warranted. Finally, the lack of ethnoracial diversity in ***The 90* + *Study*** limits the generalizability of our findings to more diverse populations.

## Conclusions

In one of the largest and most well-characterized autopsy cohorts of the oldest old, we found that in the oldest old with intermediate or high ADNC, 22–33% maintained normal cognition and 67–78% had dementia. The likelihood for the oldest old with intermediate/high ADNC to have normal cognition was higher if they had college education or higher and hypertension. It was lower for participants with transient ischemic attacks. Several non-ADNCs were related to higher likelihood of dementia, including LBD, LATE-NC, HS, and a high number of non-ADNCs. These findings highlight the role of health and lifestyle factors that contribute to maintaining normal cognition in the presence of intermediate/high AD neuropathology. Findings also suggest that non-ADNCs may be important contributors to dementia in those with intermediate/high ADNC. Our findings could inform interventions to target specific health conditions and neuropathologic changes that lower the likelihood of remaining cognitively intact in the presence of ADNC.

## Supplementary Information

Below is the link to the electronic supplementary material.

Supplementary file1 (DOCX 19 KB)

## Data Availability

The dataset used for the current study is available from the corresponding author on reasonable request.
