# Supplementary material for: Contribution of health history and neuropathologic changes to the likelihood of dementia in those with intermediate/high Alzheimer’s pathology: findings from The 90 + Study
Source: Acta Neuropathol. 2026 Apr 6;151(1):36. doi: 10.1007/s00401-026-03010-9 (PMC13053464; doi:10.1007/s00401-026-03010-9)
Supplement: Supplementary file 1 — Supplementary file1 (DOCX 19 KB) [file 401_2026_3010_MOESM1_ESM.docx]

**Supplementary materials**

| **Supplementary Table 1. Frequencies of neuropathologic changes using original categorization for intermediate/ high and high ADNC by resilience status** | | | | |
| --- | --- | --- | --- | --- |
|  | **Intermediate/ High ADNC (N=235)** | | **High ADNC (N=114)** | |
|  | **Resilient**  **(Normal Cognition) (N = 77)** | **Non-resilient**  **(Dementia) (N = 158)** | **Resilient**  **(Normal Cognition) (N = 25)** | **Non-resilient**  **(Dementia) (N = 89)** |
|  | **N (%)** | | | |
| **AD Severity Score** |  |  |  |  |
| Intermediate | 052 (68) | 069 (44) | 0 | 0 |
| High | 025 (33) | 089 (56) | 25 (100) | 89 (100) |
| **Thal Phase** |  |  |  |  |
| 1 | 002 (3) | 002 (1) | 0 | 0 |
| 2 | 004 (5) | 002 (1) | 0 | 0 |
| 3 | 024 (31) | 033 (21) | 0 | 0 |
| 4 | 040 (52) | 076 (48) | 18 (72) | 49 (55) |
| 5 | 007 (9) | 045 (29) | 7 (28) | 40 (45) |
| **Tangle Braak Stage** |  |  |  |  |
| III | 014 (18) | 009 (6) | 0 | 0 |
| IV | 035 (46) | 048 (30.4) | 0 | 0 |
| V | 027 (35) | 073 (46) | 24 (96) | 62 (70) |
| VI | 001 (1) | 028 (18) | 1 (4) | 27 (30) |
| **CERAD** |  |  |  |  |
| None | 000 | 001 (1) | 0 | 0 |
| Sparse | 011 (14) | 016 (10) | 0 | 0 |
| Moderate | 013 (17) | 021 (13) | 0 | 2 (2) |
| Frequent | 053 (69) | 120 (76) | 25 (100) | 87 (98) |
| **LBD** |  |  |  |  |
| None | 060 (78) | 095 (60) | 20 (80) | 52 (58) |
| Olfactory | 005 (7) | 015 (10) | 01 (4) | 8 (9) |
| Lower raphe nuclei | 000 | 005 (3) | 00 | 2 (2) |
| Substantia nigra | 005 (7) | 010 (6) | 01 (4) | 7 (8) |
| Temporal mesocortex | 000 | 001 (1) | 00 | 1 (1) |
| Temporal neocortex | 005 (7) | 011 (7) | 01 (4.0) | 7 (8) |
| MFG | 002 (3) | 021 (13) | 02 (8.0) | 12 (14) |
| **HS** |  |  |  |  |
| No | 071 (92) | 127 (80) | 24 (96) | 71 (80) |
| Yes | 006 (8) | 031 (20) | 1 (4) | 18 (20) |
| **LATE-NC** |  |  |  |  |
| None | 055 (71) | 078 (49) | 19 (76) | 40 (45) |
| Amygdala | 008 (10) | 013 (8) | 01 (4) | 08 (9) |
| Hippocampus | 013 (17) | 061 (39) | 04 (16) | 38 (43) |
| Cortex | 001 (1) | 006 (4) | 01 (4) | 03 (3) |
| **Arteriosclerosis** |  |  |  |  |
| None | 010 (13) | 022 (14) | 01 (4) | 13 (15) |
| Mild | 020 (26) | 042 (27) | 04 (16) | 26 (29) |
| Moderate | 046 (60) | 093 (59) | 20 (80) | 49 (55) |
| Severe | 001 (1) | 001 (1) | 00 | 01 (1) |
| **Atherosclerosis^a^** |  |  |  |  |
| None | 018 (23) | 031 (20) | 5 (20) | 16 (18) |
| Mild | 032 (42) | 063 (41) | 11 (44) | 37 (43) |
| Moderate | 025 (33) | 048 (31) | 8 (32) | 27 (31) |
| Severe | 002 (3) | 013 (8) | 1 (4) | 7 (8) |
| **CAA** |  |  |  |  |
| None | 028 (36) | 049 (31) | 7 (28) | 21 (24) |
| Mild | 011 (14) | 024 (15) | 2 (8) | 14 (16) |
| Moderate | 027 (35) | 060 (38) | 9 (36) | 33 (37) |
| Severe | 011 (14) | 025 (16) | 7 (28) | 21 (24) |
| **MVL** |  |  |  |  |
| 0 | 063 (82) | 120 (76) | 24 (96) | 72 (81) |
| 1 | 010 (13) | 022 (14) | 1 (4) | 9 (10) |
| 2 | 003 (4) | 007 (4) | 0 | 4 (5) |
| 3 | 001 (1) | 009 (6) | 0 | 4 (5) |
| **Non-ADNC** |  |  |  |  |
| 0 | 002 (3) | 004 (3) | 1 (4) | 1 (1) |
| 1 | 020 (26) | 017 (11) | 5 (20) | 10 (11) |
| 2 | 035 (46) | 057 (36) | 10 (40) | 29 (33) |
| 3 | 016 (21) | 050 (32) | 8 (32) | 27 (30) |
| 4 | 004 (5) | 022 (14) | 1 (4) | 18 (20) |
| 5 | 000 | 006 (4) | 0 | 3 (3) |
| 6 | 000 | 002 (1) | 0 | 1 (1) |
| ^a^3 participants miss atherosclerosis data. ADNC=Alzheimer’s disease neuropathologic change; CERAD=Consortium to Establish a Registry for Alzheimer’s Disease; LBD= Lewy bodies disease; HS= Hippocampal sclerosis; LATE-NC=Limbic Predominant Age-Related TDP-43 Encephalopathy Neuropathologic Change ; CAA=Cerebral amyloid angiopathy; MVL=Microvascular lesions. | | | | |
